# Pharmacokinetic Properties of Arsenic Species after Intravenous and Intragastrical Administration of Arsenic Trioxide Solution in Cynomolgus Macaques Using HPLC-ICP-MS

**DOI:** 10.3390/molecules24020241

**Published:** 2019-01-10

**Authors:** Qiaoli Shi, Mingyan Ju, Xiaoxia Zhu, Hui Gan, Ruolan Gu, Zhuona Wu, Zhiyun Meng, Guifang Dou

**Affiliations:** 1 Department of Pharmaceutical Sciences, Beijing Institute of Radiation Medicine, 27 Taiping Road, Haidian District, Beijing 100850, China; qiaoli0212@126.com (Q.S.); 13681022512@163.com (X.Z.); ganh2003@163.com (H.G.); gurl311@126.com (R.G.); nirvasen@sina.com (Z.W.); 2 Shanghai Yao Jian Medical Biotechnology Limited Company. National University of science and technology, University of Shanghai for Science and Technology, 128 Xiangyin Road, Yangpu District, Shanghai 200433, China; 18601113236@126.com

**Keywords:** arsenic trioxide, HPLC-ICP-MS, arsenic species, cynomolgus macaque, plasma, pharmacokinetic, bioavailability

## Abstract

A rapid and sensitive method was established for arsenic (As) speciation based on high performance liquid chromatography coupled to inductively coupled plasma mass spectrometry (HPLC-ICP-MS). This method was validated for the quantification of four arsenic species, including arsenite (As^III^), arsenate (As^V^), monomethylarsonic acid (MMA^V^) and dimethylarsinic acid (DMA^V^) in cynomolgus macaque plasma. Separation was achieved in just 3.7 min with an alkyl reverse phase column and highly aqueous mobile phase containing 20 mM citric acid and 5 mM sodium hexanesulfonate (pH = 4.3). The calibration curves were linear over the range of 5–500 ng·mL^−1^ (measured as As), with *r* > 0.99. The above method was validated for selectivity, precision, accuracy, matrix effect, recovery, carryover effect and stability, and applied in a comparative pharmacokinetic study of arsenic species in cynomolgus macaque samples following intravenous and intragastrical administration of arsenic trioxide solution (0.80 mg·kg^−1^; 0.61 mg·kg^−1^ of arsenic); in addition, the absolute oral bioavailability of the active ingredient As^III^ of arsenic trioxide in cynomolgus macaque samples was derived as 60.9 ± 16.1%.

## 1. Introduction 

Despite the well-known toxicity of arsenic (As), arsenic trioxide (ATO) has been used for centuries. In the 18th century, Doctor Thomas Fowler developed a solution of arsenic trioxide in potassium bicarbonate (1% *w*/*v*) for the treatment of a variety of infectious and malignant diseases, including asthma, epilepsy, eczema and psoriasis [1,2]. In the 1970s, Chinese scholars found that ATO has a significant positive effect on acute promyelocytic leukemia (APL), with much less adverse side effects compared with conventional chemotherapeutic drugs [3,4]. Meanwhile, ATO was approved by the Food and Drug Administration (FDA) for use as a first-line anticancer drug for the treatment of relapsed or refractory APL in 2000 [5]. It was found that the rate of complete remission in relapsed patients could be as high as 92% with ATO alone [6]. Preclinical studies have shown that arsenic has multiple mechanisms of action, including apoptosis and leukemia cell differentiation [7,8,9,10]. Thus, it has a broad prospect of clinical application.

The typical ATO regimen is 4–8 weeks of intravenous infusion; however, there are many obstacles to intravenous administration, particularly over such a long term. Potential problems include inconvenience, risk from the injection, cardiac toxicity, cost of long-term injection and hospitalization management, which jointly make the treatment extremely challenging. Oral formulation of ATO may solve these issues and is easier to manage; the potential toxicity of arsenic can be relieved by using an appropriate dosage, while adverse effects on the heart can be greatly reduced [11,12,13,14]. However, oral administration is currently not widely used. The University of Hong Kong has successfully developed an oral formulation of ATO. A clinical study showed that oral ATO is highly effective in relapsed APL, with efficacy comparable to that of intravenous ATO [15]. However, there is no comprehensive report on the pharmacokinetics of arsenic species after treatment with oral ATO.

**Figure 1:**
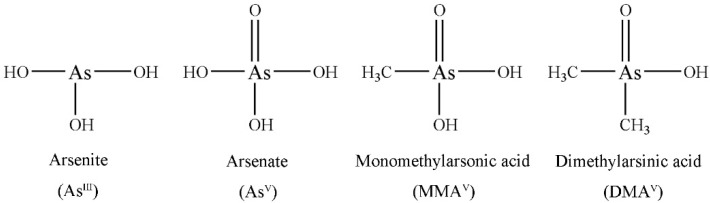
Chemical structures of the four arsenic species.

Arsenic (As) can be converted into different chemical forms or species upon entering the body, including inorganic arsenic [arsenite (As^III^), arsenate (As^V^)] and organic forms [monomethylarsonic acid (MMA^V^), dimethylarsinic acid (DMA^V^), arsenobetaine (AsB), arsenocholine (AsC) and so on]. It is widely acknowledged that the toxicological and biochemical properties of trace elements depend to a large extent on species. Inorganic arsenic is more toxic than the organic counterpart, while AsB, AsC and other arsenosugars are considered to be non-toxic [16]. Arsenite has a direct affinity to the sulfhydryl group, inducing structural modifications in proteins and enzyme inactivation [17,18]. Arsenate is similar to phosphate in biochemical properties, and can impair ATP synthesis [19]. MMA^V^ is an intermediate metabolite of arsenic metabolism, with certain toxicity [20]. DMA^V^ is the predominant methylated metabolite, which is considered a tumor promoter [21,22,23]. Therefore, there are high concerns about As^III^, As^V^, MMA^V^ and DMA^V^ (Figure 1) in clinical studies. 

High performance liquid chromatography (HPLC) coupled to inductively coupled plasma mass spectrometry (ICP-MS) is a widely used technique in elemental speciation of compounds such as arsenic, chromium, mercury, selenium and antimony [24,25,26,27,28]. Arsenic species in human urine have been analyzed; although urine is a commonly accepted indicator of arsenic exposure [29,30,31], arsenic in blood may indicate exposure to recent arsenic-containing drugs. In addition, arsenic mostly binds to blood cells [32], and exists in free state in plasma for distribution to various body tissues; therefore, it is necessary to detect arsenic species in plasma. However, the pharmacokinetic data of arsenic species in plasma are presently limited [33]. 

Oral and intravenous administration of ATO are two different drug delivery routes that may generate different types and concentrations of arsenic species in the body, resulting in toxicity differences. In this study, we developed and validated an HPLC-ICP-MS method to simultaneously quantitate the concentrations of As^III^, As^V^, MMA^V^ and DMA^V^ in cynomolgus macaque plasma samples within 3.7 min. This method was successfully used to assess the pharmacokinetic properties of As^III^, As^V^, MMA^V^ and DMA^V^ in cynomolgus macaque plasma following intravenous and intragastrical administration of arsenic trioxide solution (0.80 mg·kg^−1^; 0.61 mg·kg^−1^ of arsenic). The absolute bioavailability of the active ingredient As^III^ of ATO was determined. The present findings provide novel insights into the development of oral ATO formulation and a reliable scientific basis for clinical precision medicine.

## 2. Results and Discussion

### 2.1. Method Development

The method was assessed for compliance with the bioanalytical method validation of the European Medicines Agency (EMA) [34] and referred to the Chinese Food and Drug Administration (CFDA) [35].

#### 2.1.1. Optimization of the HPLC-ICP-MS Method

**Figure 2:**
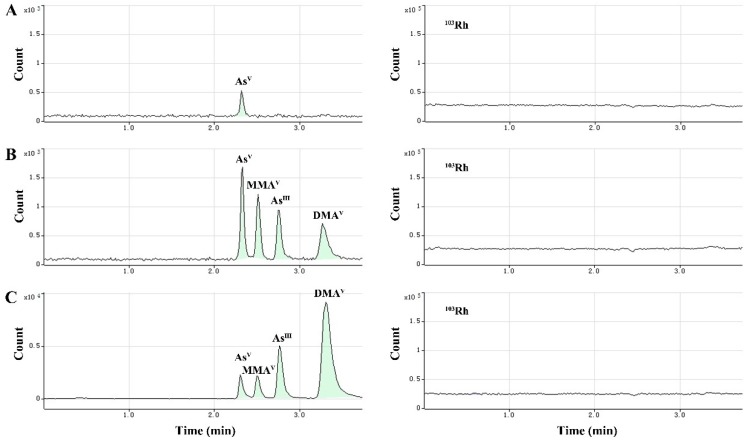
Typical chromatograms showing baseline separation of As species, including As^V^ (retention time (RT) = 2.30 min), MMA^V^ (RT = 2.51 min), As^III^ (RT = 2.77 min) and DMA^V^ (RT = 3.32 min), and Rh internal standard (right-side chromatograms). (**A**) Blank plasma sample, (**B**) blank plasma sample spiked with the analytes at the lower limit of quantitation (LLOQ), (**C**) cynomolgus macaque plasma sample after dosing of arsenic trioxide solution.

In this study, we selected a reverse phase column to separate the four As species. The method used alkyl sulfonate and arsenide of anionic form to develop ion-pair compounds, which showed hydrophobicity and could be retained and separated by the reverse phase column. In HPLC-ICP-MS, the mobile phase of HPLC should not only be suitable for separation but also compatible with the ICP-MS system. Isocratic elution was used with a mobile phase composed of aqueous solutions of 20 mM citric acid and 5 mM sodium hexanesulfonate, adjusted to pH 4.3 with 2 M sodium hydroxide. This mobile phase achieved satisfactory separation of the four As species within 3.7 min. In practical applications, the mobile phase and plasma samples containing high amounts of total dissolved solids (TDS) introduced into the ICP-MS system could cause the matrix to accumulate on the sampling cone, resulting in instrument signal drift and gradual loss of sensitivity in long-term analysis. To correct this, post column online internal standard (IS, Rhodium—^103^Rh, 1.0 µg·mL^−1^) addition was performed via a T-type three-way valve (two inlets and one outlet). This valve allows on-line mixing of the sample eluate and internal standard solutions. With post-column addition of the ISTD, the signal of the internal standard appeared at a relatively constant level, rather than a chromatographic peak. As a multi-element detector, ICP-MS can determine Rh ISTD and As analyte signals almost simultaneously; thus, point-to-point internal standard correction was used to eliminate any drift in arsenic signals. Figure 2 shows the stable IS (^103^Rh) signal during the analysis, which demonstrates the good stability of this method. 

For detecting arsenic in plasma samples, ^75^As^+^ may show spectral overlap with ^40^Ar^35^Cl^+^ and ^40^Ca^35^Cl^+^ polyatomic ions, which would yield false results for As amounts. In this work, the helium collision cell mode was employed to effectively remove such plasma-based and matrix-based polyatomic interferences, allowing an accurate determination of As signals in the complex plasma sample matrix. 

#### 2.1.2. Sample Preparation

In this study, we found As^III^ transformation into As^V^, which may be due to As^III^ instability in the aerobic environment. To prevent species inter-conversion during the process of sample collection and treatment, addition of 0.05 g solid ammonium sulfate per 100 µL of plasma was found to have a clear and obvious inhibitory effect on As^III^ conversion to As^V^. We divided the experiment into two groups, including the As^III^ and As^V^ groups (five parallel samples per group). In the As^III^ group, 10 μL standard solution of As^III^ with or without ammonium sulfate (0.05 g) was mixed with 100 μL of blank cynomolgus macaque plasma (As^III^ at a final concentration of 400 ng/mL). In the As^V^ group, the same procedure was followed except that As^III^ was substituted by As^V^. All samples were processed by the method described in “3.4 Sample preparation”. In the As^III^ group, As^V^ was detected with or without ammonium sulfate, and comprised 0.6 ± 0.04% and 11.1 ± 0.69% of the sum of inorganic arsenic (As^III^ and As^V^), respectively. This indicated that plasma supplemented with ammonium sulfate could clearly inhibit the formation of As^V^. In the As^V^ group, the other three arsenic species (As^III^, MMA^V^ and DMA^V^) were not detected with or without ammonium sulfate, suggesting that ammonium sulfate addition or not would not change As^V^ during sample processing. According to the above results, we hypothesized that ammonium sulfate inactivates certain enzymes in cynomolgus macaque plasma, thereby inhibiting the conversion of As^III^ to As^V^. 

A methanol and acetonitrile (7:3, *v*/*v*) mixture was used for protein precipitation. Addition of organic solvents has also been found to improve ionization, and therefore, increase signals for poorly-ionized elements such as arsenic. However, high levels of organic solvent in samples can lead to carbon (soot) deposition on the sampling cone. This would affect signal stability and intensity, as well as the life expectancy of the cone. When the organic solvent content exceeds a certain level, operating conditions must be changed to avoid soot deposition and the possibility of plasma signals being extinguished. For example, a narrower torch injector, lower spray chamber temperature and addition of a small oxygen flow to the carrier gas can all allow 100% volatile solvent solutions to be run. However, in this work, we avoided the problems associated with organic solvents by drying the samples under nitrogen at 60 °C for solvent evaporation. The residue was then re-dissolved and concentrated in the mobile phase ready for sample analysis.

### 2.2. Analytical Method Validation

#### 2.2.1. Selectivity

The selectivity of the method for each As species was assessed with blank plasma samples from six individual test subjects. No interfering peaks were observed with the same retention times (RTs) as As^III^, MMA^V^ and DMA^V^, respectively. However, a peak had the same RT as As^V^. The total arsenic content of feed and drinking water provided to experimental animals was in line with Chinese national standards [36,37]. However, a peak presumed to be As^V^ was detected in the six blank cynomolgus macaque plasma samples, accounting for 33.3 ± 4.2% of the lower limit of quantitation (LLOQ, 5 ng·mL^−1^). Therefore, we hypothesized that As^V^ occurs as an endogenous component in cynomolgus macaques. In experiments assessing cynomolgus macaques, we also observed a small exogenous contribution to total As^V^. This was most likely derived from the fruits and vegetables used for diet supplementation every day, because the daily diet was the main source of inorganic arsenic exposure prior to the administration of ATO. The total arsenic of fruits and vegetables has been reported to include a large proportion of inorganic arsenic [38]. Meanwhile, As^V^ is more stable than As^III^ in an aerobic environment, thus, inorganic As (iAs) in the environment is mostly in the form of As^V^ [39,40,41].

The typical chromatograms obtained from blank samples, spiked plasma samples with analytes at the LLOQ, and plasma samples after dosing of arsenic trioxide solution are presented in Figure 2A–C. The retention times of As^III^, As^V^, MMA^V^ and DMA^V^ were 2.77 min, 2.30 min, 2.51 min and 3.32 min, respectively.

#### 2.2.2. Linearity and Sensitivity

The calibration standards of As^III^, As^V^, MMA^V^ and DMA^V^ ranged from 5-500 ng·mL^−1^ (count as As). The typical calibration equations were: As^III^, y = 425.4x + 264.7 (*r* = 0.9998); As^V^, y = 428.2x + 917.0 (*r* = 0.9999); MMA^V^, y = 470.8x + 178.7 (*r* = 0.9999); DMA^V^, y = 487.9x + 384.1 (*r* = 0.9999). The LLOQ of all four analytes was 5 ng·mL^−1^ (count as As). The results showed a good linearity over the concentration range of interest.

#### 2.2.3. Accuracy and Precision

The intra- and inter-day accuracy and precision were determined by LLOQs and quality control (QC) samples at low, medium and high concentrations. The accuracy and precision data are summarized in Table 1. The intra- and inter-day precision (relative standard deviation, RSD%) of the four arsenic species was less than 12.7%; accuracy (relative error, RE%) ranged from −6.8% to 4.7%, demonstrating that the method had good accuracy and reproducibility. 

#### 2.2.4. Matrix effects and Recovery

Matrix effect and recovery data are presented in Table 2. The matrix effects of As^III^, As^V^, MMA^V^ and DMA^V^ were in the ranges of 99.7–101.9%, 93.5–98.4%, 95.9–97.0% and 98.6–101.3%, respectively. RSD (%) values for the four analytes at two QC concentrations were ≤3.2%. These findings demonstrated that there was no obvious ion suppression or enhancement from the plasma matrix in this approach.

The overall recoveries of As^III^, As^V^, MMA^V^ and DMA^V^ were in the ranges of 36.5–38.7%, 33.7–37.3%, 39.5–39.8% and 39.5–41.8%, respectively. RSD (%) values for the four analytes were ≤3.8%. The results met the criteria that RSD (%) should not exceed 15%. It has been reported that the total arsenic content in blood cells is much higher than that in plasma [32,42], suggesting that arsenicals bind more to blood cells after entering the body, and the major arsenic in blood cells is likely bound to hemoglobin [43,44]. Additionally, arsenicals have the ability to bind to plasma proteins in animals [45,46], thus, the low recovery rate in this experiment may be caused by the combination of arsenic species and plasma proteins.

#### 2.2.5. Carryover Effects

Carryover effects for As^III^, MMA^V^ and DMA^V^ were less than 20% of the LLOQ, thus, they met the method requirements. However, As^V^ was measured at 34.1 ± 5.3% of the LLOQ, exceeding the acceptable standard. In “2.2.1 Specificity”, we mentioned that As^V^ was an endogenous component in cynomolgus macaque plasma, accounting for 33.3 ± 4.2% of the LLOQ, which is very close to its carryover value. Thus, the measured “carryover” can be explained by the endogenous As in blank plasma samples. Moreover, As^V^ is an inactive component among metabolites of arsenic trioxide. RSD% and RE% of accuracy and precision for As^V^ were less than 15% (Section 2.2.3), which met the requirements of the EMA and CFDA, demonstrating that As^V^ quantitation was reliable.

#### 2.2.6. Stability

Stability data are displayed in Table 3. The results showed that the four species of arsenic in blank plasma were stable at room temperature for 8 h, after three freeze and thaw cycles, and at −80 °C for 172 days. The processed samples were stable at 4 °C for 24 h, and after loading onto the auto-sampler for 24 h. Furthermore, the stock solution of arsenic species was stable at 4 °C for 50 days.

### 2.3. Pharmacokinetic Profiles of Arsenic Species in Mammals

This method was successfully applied to the pharmacokinetic study of the four arsenic species in mammalian plasma after intravenous (i.v.) and intragastrical (i.g.) administration of arsenic trioxide solution at a single dose of 0.80 mg/kg (equivalent to 0.61 mg As/kg), respectively.

**Figure 3:**
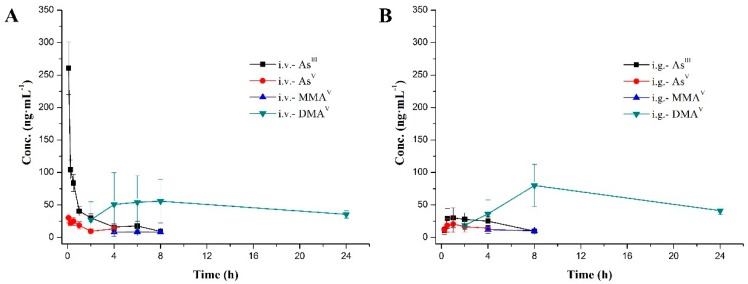
Mean plasma concentration-time curves of the four arsenic species after administration (**A**. intravenous; **B**. intragastrical) at a single dose of 0.80 mg·kg^−1^ (0.61 mg·kg^−1^ of arsenic). Data are mean ± SD (*n* = 10).

The plasma concentration-time profiles of the four species after intravenous and intragastrical administration of arsenic trioxide solution are shown in Figure 3, and mean pharmacokinetic parameters are summarized in Table 4. As shown in Figure 3, As^III^ is an effective anti-cancer compound and could be detected within 8 h of both intravenous and intragastrical administration. As^V^ was detected within 4 h. MMA^V^ as an intermediate metabolite appeared at 4–8 h, and remained at a stable and relatively low level in both groups. As the predominant metabolite, DMA^V^ was found at high levels, starting from 2 h after dosing through 24 h in both groups.

As^III^ reached maximum plasma concentrations (C_max_) of 260.7 ± 40.7 and 40.8 ± 8.4 ng·mL^−1^, AUC_last_ values of 243.1 ± 51.8 and 148.1 ± 39.0 h·ng·mL^−1^, following intravenous and intragastric administration of arsenic trioxide solution at a single dose of 0.80 mg/kg (equivalent to 0.61 mg As/kg), respectively. The time of maximum concentration (T_max_) after intravenous administration was the first time point (0.083 h), and the intragastrical administration was 1.7 ± 1.3 h. As^V^, reached maximum plasma concentrations (C_max_) of 30.5 ± 7.7 ng·mL^−1^ (i.v.) and 24.5 ± 10.9 ng·mL^−1^ (i.g.), with AUC_last_ values of 55.9 ± 23.7 h·ng·mL^−1^ (i.v.) and 172.6 ± 139.9 h·ng·mL^-1^ (i.g.), respectively. As for MMA^V^, AUC_last_ values of the i.v. and i.g. group were both lower compared with those of the other three species. The pharmacokinetics (PK) parameters of DMA^V^ after i.v. were similar to i.g. counterparts; the mean residence time (MRT_last_) was the longest among the four arsenic species.

In addition, absolute oral bioavailability of As^III^, which was estimated by dividing AUC_i.g._ by AUC_i.v._, was 60.9 ± 16.1%, indicating that orally administered drug could be well absorbed. Arsenic trioxide existed in the solution as As^III^, which is the pharmacologically active species of arsenic trioxide, while As^V^, MMA^V^ and DMA^V^ are all metabolites of As^III^. Therefore, ATO bioavailability assessment using As^III^ levels is more accurate than employing total arsenic content.

From the above, the durations of the parent drug (As^III^) and its three metabolites (As^V^, MMA^V^ and DMA^V^) in vivo after intragastrical administration were relatively consistent with intravenous administration. The most striking difference was that the instantaneous plasma concentration of the parent drug (As^III^) after intravenous administration was very high (C_max_, 260.7 ± 40.7 ng·mL^−1^, i.v.), while that after intragastrical administration was relatively low, but was very stable in the body (C_max_, 40.8 ± 8.4 ng·mL^−1^, i.g.). High instantaneous concentration results in cardiac QTc interval prolongation [14]; oral administration of As^III^ may relieve this side effect. Therefore, oral formulations have similar therapeutic effects compared with intravenous products, but oral administration is more convenient and safer.

Generally speaking, biotransformation of inorganic arsenic involves reduction and methylation reactions in mammals. After ingestion, As^V^ is first reduced to As^III^, which is methylated to MMA^V^; in turn, MMA^V^ is reduced to MMA^III^, which is methylated to DMA^V^, which is finally reduced to DMA^III^ [41,47,48]. Arsenic metabolism usually begins with As^V^, which is more stable than As^III^; indeed, As^V^ is widely found in aerobic ecological environment [39,40,41]. In Figure 3, As^III^ and As^V^ were detected at the first time point after i.v. and i.g. administration (i.v., 0.083 h; i.g., 0.25 h). We hypothesized that a small amount of As^III^ is rapidly transformed into As^V^ after ATO administration in the blood or gastrointestinal tract, that is to say, in certain conditions, the first step in the metabolism of inorganic arsenic is a reversible reaction. However, the metabolic enzyme promoting such reaction remains unknown, which deserves further exploration.

The results also showed elevated standard deviation (SD) values for the four arsenic species. This suggested that there was a marked inter-individual variation in arsenic metabolism; moreover, there is a considerable species variation in arsenic methylation. The main reason for differences in inorganic arsenic methylation between species and individuals is gene polymorphism [40,49,50,51], which plays an important role in the regulation of enzymes involved in iAs biotransformation. Furthermore, the intestinal flora is worth assessing in relation to arsenic metabolism [52,53,54]. We performed statistical analysis of male-female differences in the seven pharmacokinetic parameters (T_max_, C_max_, AUC_last_, AUC_INF_obs_, CL__obs,_ Vss__obs_ and MRT_last_) for the four arsenic species by two-tailed t-test (IBM SPSS). There were no significant differences between males and females (*p* > 0.05) for the seven pharmacokinetic parameters in the same arsenic species and dose group, except that the pharmacological parameters C_max_ and AUC_last_ (*p* < 0.05) of the main metabolite DMA^V^ after intragastrical administration were significantly different. Moreover, males had significantly higher levels than females (C_max_, 1.7 times; AUC_last_, 1.5 times). In the intravenous group, although there were no statistical differences between males and females in C_max_ and AUC_last_ of DMA^V^ (*p* > 0.05), the average value of males in these two parameters were slightly higher than that of females (C_max_, 1.4 times; AUC_last_, 1.3 times). It has been reported that there was a difference in gender in the amount of DMA^V^ [55,56]; some authors reported no differences [57]. In this study, the C_max_ and AUC_last_ of DMA^V^ after intravenous and intragastrical administration of ATO were slightly higher in males than that of females. We believe that slight gender differences may have an effect on arsenic methylation metabolism. The specific reasons deserve further study.

Beagle dogs were first chosen for the study, but because of strong stimulation by ATO of the gastrointestinal tract, which is especially sensitive in beagle dogs [58], vomiting was so severe that the dosage was not accurate; therefore, we needed another animal model. Some nonhuman primates, including the marmoset, chimpanzee and tamarin monkey [59,60,61,62], could not methylate inorganic arsenic, and the process of inorganic arsenic methylation acts as a detoxification mechanism [40,60]. No literature has been reported on inorganic arsenic metabolism in the cynomolgus macaque. In this study, methylated metabolites (MMA^V^ and DMA^V^) were detected in the plasma of cynomolgus macaques after intravenous and intragastrical administration of ATO, indicating that cynomolgus macaques have methylation ability as humans [63,64,65]. This study provides insights into arsenic metabolism in non-human primates, and implies that the cynomolgus macaque is an ideal animal model for assessing inorganic arsenic metabolism, which has considerable guiding significance for oral administration of ATO in humans in the future.

In preliminary experiments in the cynomolgus macaque, we quantified five arsenic species in plasma (As^III^, As^V^, MMA^V^, DMA^V^ and AsB) by HPLC-ICP-MS. The results showed that AsB amounts at any time point in plasma were lower than the LLOQ (5 ng·mL^−1^), and the AsB peak was at the end of the chromatogram (As^V^, RT = 2.24 min; MMA^V^, RT = 2.48 min; As^III^, RT = 2.74 min; DMA^V^, RT = 3.24 min; AsB, RT = 3.83 min); AsB is usually considered to be non-toxic. For the above reasons, AsB data were not shown in this study.

## 3. Materials and Methods

### 3.1. Chemicals and Reagents

Arsenic trioxide solution was manufactured by Jinan Chuan Cheng Pharmaceutical R&D Co., Ltd. (Jinan, China). Arsenic acid, arsenious acid, monomethylarsonic acid and dimethylarsinic acid solutions were purchased from NIMC (National Institute of Metrology China, Beijing, China). ICP-MS Internal Standard Mixture and ICP-MS Stock Tuning Solution were purchased from Agilent Technologies Co., Ltd. (Santa Clara, CA, USA). High purity citric acid (≥99.5%), sodium hexane-sulfonate (≥98%) and sodium hydroxide (≥99.99%) were purchased from Sigma Aldrich Chemicals (St. Louis, MO, USA). Methanol, acetonitrile, and isopropanol were obtained from Thermo Fisher Scientific Inc., (Waltham, MA, USA). Ammonium sulfate was from SCRC (Sinopharm Chemical Reagent Co., Ltd., Beijing, China). Concentrated nitric acid (68.8–69.8%) was purchased from DUKSAN (KYUNGKIDO. ANSAN, Korea). Sodium chloride injection (500 mL:4.5 g) was obtained from Cisen Pharmaceutical Co., Ltd. (Jining, China). Cynomolgus macaque plasma was supplied by Xieerxin Biology Resource (Beijing, China). Deionized water with a resistivity of 18.2 MΩ·cm^−1^ was prepared with a Milli-Q^®^ Advantage A10 (Millipore Corp., Billerica, MA, USA).

### 3.2. Instruments and HPLC-ICP-MS Conditions

An Agilent 1260 HPLC system comprising a quaternary pump, autosampler and vacuum degasser was used for As species separation, coupled to an Agilent 7700x ICP-MS (Agilent, Santa Clara, CA, USA) for detection. The column exit was simply connected to the nebulizer of the ICP-MS by PFA tubing. The reverse phase column Agilent ZORBAX SB-Aq (4.6 mm id × 250 mm, 5 μm) was maintained at room temperature throughout the analysis. The mobile phase was 20 mM citric acid and 5 mM sodium hexanesulfonate, adjusted to pH 4.3 with 2 M sodium hydroxide. The injection volume was 10 µL; a flow rate of 1.0 mL·min^−1^ was adopted, with a run time of 3.7 min.

An Agilent 7700x ICP-MS with an Octopole Reaction System (ORS^3^, Santa Clara, CA, USA) collision/reaction cell (CRC) was used for As species detection. The 7700 ICP-MS was fitted with a standard Micromist nebulizer and a Scott spray chamber (temperature controlled at 2 °C). Typical operating parameters for ICP-MS were: RF power, 1550 W; carrier gas flow rate, 0.8 L·min^−1^; makeup gas flow rate, 0.4 L·min^−1^; sampling depth; 8.0 mm.

Helium cell gas was used to rule out potential polyatomic ion interference (^40^Ar^35^Cl^+^ or ^40^Ca^35^Cl^+^) on ^75^As^+^. The helium cell gas flow rate was 4.0 mL·min^−1^. ^103^Rh was used as post-column online internal standard to correct long-term signal drift caused by the plasma matrix.

### 3.3. Preparation of Stock and Working Solutions

A mixed stock solution of As^III^, As^V^, MMA^V^ and DMA^V^ was obtained by appropriate dilution of standard solutions with purified water, to a final concentration of 5000 ng·mL^−1^ (measured as As) for As^III^, As^V^, MMA^V^ and DMA^V^. The stock solution was stored at 4 °C, and working standard solutions were freshly prepared before use. An internal standard working solution (1.0 µg·mL^−1^) was prepared in isopropanol and 5% nitric acid (5:95, *v*/*v*) in a final volume of 100 mL. The internal standard was also stored at 4 °C.

### 3.4. Sample Preparation

Plasma samples were thawed to room temperature before use. A total of 0.05 g ammonium sulfate was added into each empty centrifuge tube before use to prevent As^III^ conversion to As^V^. Then, 100 μL plasma sample was added to the tube and vortexed for 2 min. Next, 10 µL water and 200 µL methanol/acetonitrile (7:3, *v*/*v*) were added for protein precipitation. The mixture was centrifuged at 12,000 rpm for 15 min at 4 °C after vortex-mixing for 2 min. The supernatant (120 μL) was transferred to a 1.5 mL Eppendorf tube, dried under nitrogen at 60 °C, and dissolved in 100 μL mobile phase after cooling to room temperature. The mixture was centrifuged at 12,000 rpm for 3 min at 4 °C after vortex-mixing for 2 min. The resulting supernatant was transferred into auto-sampler vials and analyzed by HPLC-ICP-MS with 10 μL injection volume.

The calibration plasma standards were prepared by spiking blank plasma from cynomolgus macaques with the working standard solutions covering a concentration range from 5 to 500 ng·mL^−1^ for As^III^, As^V^, MMA^V^ and DMA^V^. Seven calibrators were used to cover the quantitation range: 5, 10, 20, 50, 100, 250 and 500 ng·mL^−1^. Quality control (QC) samples were prepared by the same procedures in plasma at concentrations of 8, 80 and 400 ng·mL^−1^ for As^III^, As^V^, MMA^V^ and DMA^V^.

### 3.5. Method Validation

#### 3.5.1. Selectivity

Selectivity was assessed in appropriate blank matrix samples from six individual test subjects to assess for potential interference. Any peaks present in the chromatograms should contribute <20% of LLOQ to analyte peaks.

#### 3.5.2. Linearity and Sensitivity

Seven-point calibration curves were fitted using weighted least squares linear regression of peak areas after correction with the online internal standard (^103^Rh). Each batch of calibration solutions was freshly prepared the day of use. Blank plasma samples were used to confirm the absence of interference. The LLOQ was used to determine the sensitivity of the method; the deviation should not exceed 20%.

#### 3.5.3. Accuracy and Precision

For accuracy and precision validation, six sample replicates at four different concentration levels (LLOQ, low, mid and high) were assessed on three different days. Intra- and inter-day precision (RSD%) and accuracy (RE%) were required to be within ±15%, except for the LLOQ which should be within ±20%.

#### 3.5.4. Matrix effects and Recovery

The effects of the sample matrix were evaluated by comparing peak areas in blank plasma collected from six individual test subjects. The extracted matrix was spiked with standard solutions, and peak areas were compared to those determined in pure reference standard solutions at the same concentrations. The ratio of peak areas was performed at low and high concentrations, respectively. The RSD% of area ratio at each concentration should be less than 15%.

Spike recovery in macaque plasma was determined by comparing peak areas in QC samples with those of the corresponding spiked solutions added to supernatants of the processed blank plasma samples. The recovery of analytes does not need to be 100% but should be precise and reproducible. The RSD% of the peak area ratio at each concentration should be less than 15%.

#### 3.5.5. Carryover Effect

The carryover effect was determined by injecting a blank plasma sample after the upper limit of quantification (ULOQ) standard. The peak areas of blank plasma samples run after ULOQ should be less than 20% of those measured in the LLOQ sample.

#### 3.5.6. Stability

Stability evaluation involved sample preparation, analysis and storage. The stability test used six QC samples at low and high concentrations, respectively.

Stability of QC samples was investigated after storage at room temperature for 8 h, after three complete freeze/thaw cycles (−80 °C to room temperature) or storage at −80 °C for 172 days. The processed stability samples were evaluated after storage at 4 °C for 24 h and after placement on the auto-sampler for 24 h. Stock solution stability was also evaluated after storage at 4 °C for 50 days. The relative error (RE%) for any stability test set was required to be within ±15%.

### 3.6. Pharmacokinetic Assessment

Twenty cynomolgus macaques (ten males and ten females, aged 2–6 years) were obtained from Guangxi Guidong primate development experimental Co. Ltd., Wuzhou, China. Animals were fed separately and maintained under standard laboratory conditions (temperature, 18 °C to 26 °C; relative humidity, 40% to 70%; 12/12 h light/darkness). The animals were supplemented with about 50 g of fruits or vegetables per day, unless otherwise stated. All animal experiments were conducted according to the Guide for the Care and Use of Laboratory Animals (National Research Council of the USA, 1996), and approved by the Association for Assessment and Accreditation of Laboratory Animal Care. The license for experimental animal production was SYXK (Suzhou) 2016-0024. All animal studies were approved by the Animal Ethics Committee of China Pharmaceutical University (Approval number ACU16-1086).

The twenty test animals were fasted overnight before the experiments, and randomly divided into two groups, including the intravenous (*n* = 10) and intragastrical (*n* = 10) groups, comprising half males and females. Each animal received a single dose of 0.80 mg·kg^−1^ of arsenic trioxide solution (0.5 mg/mL, equivalent to 0.61 mg·kg^−1^ of arsenic), by intragastrical (i.g.) or intravenous (i.v.) administration. Blood samples of about 0.5 mL were drawn from the animal’s forelimb or hindlimb vein into dipotassium ethylenediaminetetraacetic acid (EDTA-2K) tubes at the following time points after dosing: 0.25, 0.5, 1.0, 2.0, 4.0, 8.0, and 24 h (i.g.); 0.083, 0.25, 0.5, 1.0, 2.0, 4.0, 6.0, 8.0 and 24 h (i.v.). Plasma was collected by centrifugation at 2000 g for 10 min at 2–8 °C and stored at −70 °C until analysis.

The animal experiments were performed by JOINN Laboratories, Inc. following Good Laboratory Practice (GLP) guidelines.

### 3.7. Data Analysis

Microsoft Office Excel was used for data calculation. Origin 8.0 was used to draw graphs. ChemDraw Ultra 8.0 was used to generate the chemical structures of the four arsenic species. Agilent ICP-MS MassHunter Workstation version A 01.02 was used for data acquisition and analysis. WinNonlin Version 6.4 (Pharsight Corp., MO, USA) was used to determine the pharmacokinetic parameters of arsenical species based on a non-compartmental method. T_max_ and C_max_ were observed values, and the calculation method for AUC was linear log trapezoidal. The absolute oral bioavailability (F) of As^III^, due to the same dose of intravenous and intragastrical administration, was estimated by dividing AUC_i.g._ by AUC_i.v._ Other parameters were calculated using non-compartmental model based on the actual measured concentration values. All data were expressed in terms of the means with their standard deviation. Statistical analysis of male and female differences in each group was performed using t-test two-tailed method (IBM SPSS software). The *p* value was set as <0.05 for statistical significance.

## 4. Conclusion

In this study, we developed a rapid and stable method to simultaneously determine As^III^, As^V^, MMA^V^ and DMA^V^ amounts in cynomolgus macaque plasma. The separation technique of HPLC has the advantage of a short run time (3.7 min). The established and confirmed technique was successfully applied to perform a comparative pharmacokinetic study of arsenic species after intravenous and intragastrical administration of arsenic trioxide in cynomolgus macaques. The absolute oral bioavailability of the active component As^III^ of arsenic trioxide in cynomolgus macaque plasma was found to be 60.9 ± 16.1%, for the first time. Oral formulation of arsenic trioxide is convenient and safe, and could play an important role in long-term arsenic treatment in the future.

## Figures and Tables

**Table 1:** Detection precision and accuracy for the four analytes in cynomolgus macaque plasma (*n* = 6).

Arsenic Species	Spiked Concentration (ng·mL^−1^)	Measured Concentration (ng·mL^−1^) (Mean ± SD, *n* = 6)			Overall Mean (ng·mL^−1^)	Intra-DayRSD (%)	Inter-DayRSD (%)	RE (%)
		Batch 1	Batch 2	Batch 3				
As^III^	5.0	4.8 ± 0.1	4.8 ± 0.3	5.2 ± 0.3	5.0	5.4	12.7	−1.8
	8.1	7.8 ± 0.3	7.8 ± 0.6	8.3 ± 0.2	8.0	5.2	9.6	−1.2
	80.7	83.4 ± 2.4	77.6 ± 1.7	84.0 ± 1.7	81.6	2.4	10.6	1.2
	403.5	421.3 ± 16.0	406.8 ± 18.7	410.3 ± 10.2	412.8	3.7	4.5	2.3
As^V^	5.0	5.2 ± 0.2	4.8 ± 0.2	5.2 ± 0.3	5.1	4.5	9.5	0.5
	8.1	8.7 ± 0.5	8.3 ± 0.4	8.2 ± 0.6	8.4	5.8	7.3	4.1
	80.7	87.7 ± 3.2	84.7 ± 2.6	79.6 ± 1.7	84.0	3.1	11.8	4.2
	403.3	435.5 ± 7.7	430.0 ± 11.8	401.1 ± 9.4	422.2	2.3	10.7	4.7
MMA^V^	5.0	5.0 ± 0.2	5.0 ± 0.2	4.9 ± 0.4	4.9	5.9	4.2	−1.2
	8.0	8.3 ± 0.4	8.4 ± 0.3	8.4 ± 0.4	8.4	4.5	0.4	4.4
	80.2	83.4 ± 1.3	84.9 ± 2.6	82.0 ± 2.9	83.4	2.8	4.2	4.1
	400.8	414.7 ± 8.0	416.6 ± 5.6	396.5 ± 17.3	409.3	2.8	6.6	2.1
DMA^V^	5.0	4.6 ± 0.3	4.8 ± 0.5	4.6 ± 0.5	4.7	9.3	7.1	−6.8
	8.0	7.9 ± 0.7	8.2 ± 0.4	8.2 ± 0.5	8.1	7.1	4.1	0.9
	80.3	83.3 ± 2.4	82.5 ± 1.6	80.6 ± 1.0	82.1	2.1	4.0	2.3
	401.5	418.2 ± 4.6	417.0 ± 11.1	398.7 ± 5.1	411.3	1.8	6.5	2.4

**Table 2:** Matrix effects and recovery rates for the four analytes in cynomolgus macaque plasma.

Arsenic Species	Concentration (ng·mL^−1^)	Matrix Effect (*n* = 6)		Recovery (*n* = 6)	
		Mean ± SD (%)	RSD (%)	Mean ± SD (%)	RSD (%)
**As^III^**	8	101.9 ± 3.3	3.2	38.7 ± 0.8	2.1
	400	99.7 ± 0.6	0.6	36.5 ± 1.1	3.0
As^V^	8	98.4 ± 2.0	2.0	37.3 ± 1.3	3.6
	400	93.5 ± 1.8	1.9	33.7 ± 0.9	2.7
MMA^V^	8	97.0 ± 2.5	2.6	39.5 ± 1.5	3.8
	400	95.9 ± 1.6	1.7	39.8 ± 0.9	2.3
DMA^V^	8	101.3 ± 2.7	2.7	41.8 ± 1.2	3.0
	400	98.6 ± 1.3	1.3	39.5 ± 0.7	1.9

**Table 3:** Stability data of the four arsenicals in cynomolgus macaque plasma under different storage conditions (*n* = 6).

Storage Conditions	Concentration (ng·mL^−1^)	As^III^			As^V^			MMA^V^			DMA^V^		
		Mean ± SD (ng·mL^−1^)	RSD (%)	RE (%)	Mean ± SD (ng·mL^−1^)	RSD (%)	RE (%)	Mean ± SD (ng·mL^−1^)	RSD (%)	RE (%)	Mean ± SD (ng·mL^−1^)	RSD (%)	RE (%)
Room temperature for 8 h	8	8.4 ± 0.5	6.1	4.2	8.2 ± 0.5	5.8	1.4	8.0 ± 0.2	2.8	−0.7	8.2 ± 0.7	8.0	1.6
	400	405.9 ± 8.6	2.1	0.6	404.2 ± 13.6	3.4	0.2	394.8 ± 9.5	2.4	−1.5	394.2 ± 10.0	2.5	−1.8
Three freeze-thaw cycles	8	7.8 ± 0.2	2.5	−3.7	7.4 ± 0.3	3.5	−7.7	7.5 ± 0.2	2.4	−6.1	7.5 ± 0.1	1.6	−6.5
	400	393.2 ± 8.6	2.2	−2.6	401.9 ± 11.6	2.9	−0.4	390.5 ± 8.3	2.1	−2.6	393.4 ± 8.1	2.1	−2.0
Long term for 172 days (−80 °C)	8	7.8 ± 0.4	4.5	−3.5	7.7 ± 0.3	3.9	−4.9	7.9 ± 0.2	2.5	−1.5	7.6 ± 0.3	4.4	−5.3
	400	402.4 ± 11.7	2.9	−0.7	407.1 ± 5.5	1.3	2.1	388.3 ± 9.2	2.4	−3.7	397.6 ± 7.6	1.9	−1.0
Processed sample for 24 h (4 °C)	8	8.5 ± 0.6	7.5	4.9	8.3 ± 0.3	3.1	2.6	8.3 ± 0.1	1.2	3.6	8.1 ± 0.2	2.7	0.6
	400	404.1 ± 11.9	3.0	0.2	401.5 ± 13.4	3.3	−0.4	386.3 ± 6.1	1.6	−3.6	388.9 ± 13.6	3.5	−3.2
Autosampler for 24 h	8	7.6 ± 0.3	3.6	−5.2	7.3 ± 0.3	3.7	−10.1	7.1 ± 0.3	4.7	−11.0	7.5 ± 0.3	4.5	−6.7
	400	398.4 ± 6.5	1.6	−1.3	398.5 ± 17.3	4.3	−1.2	388.8 ± 10.0	2.6	−3.0	394.4 ± 8.9	2.2	−1.8
Stock solution for 50 days (4 °C)	8	8.0 ± 0.1	1.7	−4.2	8.0 ± 0.4	5.1	−2.3	7.7 ± 0.3	3.4	−3.4	8.2 ± 0.5	5.5	1.5
	400	402.1 ± 9.5	2.4	−3.5	406.3 ± 7.0	1.7	−0.3	405.3 ± 7.9	1.9	1.2	406.5 ± 6.2	1.5	1.0

**Table 4:** Pharmacokinetic parameters of the four arsenic species (*n* = 10).

Parameter (unit)	Mean ± SD (0.80 mg·kg^−1^, 0.61mg·kg^−1^ of Arsenic)							
	As^III^		As^V^		MMA^V^		DMA^V^	
	i.g.	i.v.	i.g.	i.v.	i.g.	i.v.	i.g.	i.v.
T_max_ (h)	1.7 ± 1.3	0.083 ± 0.0	3.6 ± 7.3	0.17 ± 0.18	8.8 ± 5.6	5.6 ± 2.1	11.2 ± 6.7	13.4 ± 9.2
C_max_ (ng·mL^−1^)	40.8 ± 8.4	260.7 ± 40.7	24.5 ± 10.9	30.5 ± 7.7	13.8 ± 6.1	9.9 ± 1.7	80.7 ± 31.8	65.1 ± 41.4
AUC_last_ (h·ng·mL^−1^)	148.1 ± 39.0	243.1 ± 51.8	172.6 ± 139.9	55.9 ± 23.7	92.6 ± 98.2	57.6 ± 9.7	1225.0 ± 364.4	1012 ± 511
AUC_INF_obs_ (h·ng·mL^−1^)	/	286.5 ± 76.8	/	76.6 ± 34.0	/	/	/	/
CL__obs_ (L·h^−1^·kg^−1^)	/	2.3 ± 0.5	/	9.9 ± 5.2	/	/	/	/
Vss__obs_ (L·kg^−1^)	/	7.4 ± 0.5	/	21.2 ± 4.5	/	/	/	/
MRT_last_ (h)	3.1 ± 0.9	2.1 ± 0.5	6.1 ± 5.5	1.4 ± 0.5	6.6 ± 3.3	3.8 ± 0.9	13.1 ± 1.0	13.2 ± 1.8
F (%)	60.9 ± 16.1							

/: The pharmacokinetic parameters cannot be calculated.

## Abbreviations

ATO	arsenic trioxide
APL	acute promyelocytic leukemia
HPLC-ICP-MS	high performance liquid chromatography coupled to inductively coupled plasma mass spectrometry
iAs	inorganic arsenic
As^III^	arsenite
As^V^	arsenate
MMA^V^	monomethylarsonic acid
DMA^V^	dimethylarsinic acid
MMA^III^	methylarsonous acid
DMA^III^	dimethylarsinous acid
i.v.	intravenous administration
i.g.	intragastrical administration
SD	standard deviation
C_max_	maximum concentration
T_max_	time of maximum concentration
AUC_last_	area under the curve from the time of dosing to the last measurable concentration
AUC_INF_obs_	AUC from dosing time extrapolated to infinity
CL__obs_	total clearance
Vss__obs_	volume of distribution at steady state
MRT_last_	mean residence time
